# Coproduction Without Youth? Closing the Participation Gap in Digital Mental Health Research

**DOI:** 10.2196/91739

**Published:** 2026-06-24

**Authors:** Charlotte Blease, Maria Tibbs, Andreas Balaskas, Shaun Liverpool, Josefin Hagström, Amanda Fitzgerald

**Affiliations:** 1 Department of Women's and Children's Studies Uppsala University Uppsala Sweden; 2 Digital Psychiatry, Department of Psychiatry Newark Beth Israel Medical Center Boston, MA United States; 3 Psychology in Education Research Centre University of York York, England United Kingdom; 4 School of Computer Science University College Dublin Dublin, Leinster Ireland; 5 Faculty of Health, Social Care and Medicine Edge Hill University Ormskirk, England United Kingdom; 6 School of Psychology University College Dublin Dublin, Leinster Ireland

**Keywords:** digital mental health, youth mental health, co-design, participatory research, patient and public involvement, generative artificial intelligence, ethics, artificial intelligence, AI

## Abstract

Young people are among the most intensive users of digital and generative artificial intelligence (GenAI)–enabled mental health tools, yet they remain underrepresented in the research and design processes that shape these technologies. Although participatory approaches such as co-design and patient and public involvement are widely endorsed as best practices, youth involvement in digital youth mental health (DYMH) research is often inconsistent, superficial, or limited to late-stage consultation. This participation gap risks producing interventions that are misaligned with young people’s lived experiences, priorities, and vulnerabilities, particularly in the context of rapidly evolving and scalable GenAI systems. This Viewpoint aims to reexamine the underlying drivers of the participation gap in DYMH research; clarify how participation is conceptualized and implemented across disciplines; and propose concrete, actionable recommendations to support more meaningful and consistent youth involvement across the research life cycle. We draw on interdisciplinary literature from digital mental health, human-computer interaction, child-computer interaction, and health research policy. Our Viewpoint integrates conceptual frameworks (eg, Lundy’s model of participation), existing reviews of co-design practices, and emerging evidence on GenAI in mental health. We adopt a life cycle–oriented perspective to examine how youth participation is distributed across stages of research and development, including problem formulation, design, implementation, and evaluation. We identify 3 interrelated drivers of the participation gap. First, conceptual and linguistic fragmentation obscures what participation entails in practice, with terms such as co-design, participatory design, user-centered design, and patient and public involvement used inconsistently across disciplines. Second, youth involvement is uneven across the research life cycle, with participation often concentrated in early ideation or usability testing but largely absent from upstream decision-making and downstream evaluation. Third, institutional barriers—including ethics review processes, consent requirements, funding constraints, and adult-centric research norms—systematically limit meaningful youth partnership. These challenges are amplified in the context of GenAI, where opaque “black box” systems, simulated therapeutic interactions, and rapid deployment cycles introduce distinct risks if youth perspectives are not integrated. We propose a set of minimum expectations to address these gaps, including explicit specification of participatory models, life cycle mapping of youth involvement, reporting of youth influence on decisions, dedicated funding for participation, proportional ethics frameworks, and mechanisms for youth-informed governance of GenAI systems. Closing the participation gap in DYMH research is both an ethical imperative and a practical necessity. Moving beyond aspirational commitments requires embedding youth participation as a standard, resourced, and accountable component of research, design, and governance. In the context of rapidly evolving digital and GenAI technologies, failure to do so risks producing interventions that are scalable but not safe, credible, or responsive to the needs of young people.

## Introduction

Young people aged 12 to 25 years are the fastest adopters of internet-enabled technologies [1,2]. As early and intensive users of smartphones, social media platforms, and, increasingly, generative artificial intelligence (GenAI) tools, they are also disproportionately exposed to both the benefits and risks associated with digital systems. Yet they can also be at the greatest risk of potential harm associated with digital tools [3,4]. These technologies now shape how young people understand mental health, seek information and help, and manage distress, often alongside or outside formal health systems. This rapid uptake presents significant opportunities to extend access to mental health support, reduce barriers to care, and meet young people where they already are. At the same time, it introduces important risks, including misinformation, overreliance on unregulated tools, data exploitation, and the reinforcement of harmful norms or vulnerabilities. Crucially, many of these risks are not incidental but reflect the adult-centric design and governance of digital systems, including limited involvement of young people in shaping priorities, safeguards, and definitions of effective support.

Despite young people’s centrality to the digital ecosystem, they remain strikingly underrepresented in the research and design of digital mental health tools intended for their use [5]. This pattern reflects a wider issue: young people are often inadequately included in health research more broadly, with implications for the relevance, acceptability, and safety of the interventions developed for them [6]. This exclusion is even more pronounced among underserved or marginalized youth, who are rarely meaningfully included in participatory digital youth mental health (DYMH) research [7]. While coproduction and patient and public involvement (PPI) are increasingly invoked as gold standards in health research, including the field of mental health, youth involvement is frequently superficial, inconsistent, or—at best—confined to late-stage consultation [8].

This raises a fundamental tension in the field: digital mental health research routinely excludes its most digitally engaged users from genuine partnership [9]. We suggest that these opportunities and challenges are particularly pronounced in DYMH. Moreover, this participation gap is particularly concerning given the sheer speed at which digital mental health tools are being developed and deployed [10]. Innovation cycles in digital health applications, especially GenAI-enabled applications, often prioritize rapid prototyping, scalability, and technical feasibility.

In practice, the need for fast turnaround in research has too often been treated as incompatible with both participation and innovation. By contrast, participatory processes and the nature of mental health care itself require time, trust, and sustained engagement. As a result, there is a risk of a growing mismatch between the full gamut of digital mental health tools being built and the lived realities, preferences, and risks experienced by young people [11]. This risk is further heightened by the proprietary nature of many industry-led GenAI tools adopted, which can further limit transparency and meaningful youth involvement.

Unlike earlier digital mental health interventions, many GenAI systems operate as opaque “black boxes,” with limited transparency regarding how outputs are generated or how risks are managed. Their conversational interfaces can simulate empathy and responsiveness [12], potentially shaping young people’s expectations of support, trust, and help-seeking behavior in ways that are not yet well understood. These characteristics introduce distinct risks: outputs may be inaccurate, biased, or inappropriate [13], while still appearing credible or supportive. In the absence of meaningful youth involvement, such systems may fail to reflect how young people interpret, use, or respond to these interactions. Youth co-design is therefore critical—not only for improving usability but also for informing the development of appropriate guardrails, safety mechanisms, and norms of interaction, as well as identifying how algorithmic biases may differentially affect young users. Involving youth in developing artificial intelligence (AI) systems for mental health care is important for creating technologies that are relevant, effective, and ethical. This perspective can help identify priorities that resonate with young people, rather than relying on researchers’ or other experts’ assumptions about such priorities [14]. Including the youth voice could also help address biases, power imbalances, and accessibility barriers in the development of AI systems that are effective, inclusive, and trustworthy [15].

Indeed, in a recent statement on digitalization and youth mental health, EuroHealthNet [16] recommends the need to “empower and include children and young people in shaping safe digital environments via cocreation.” Meaningful integration of youth perspectives, from diverse backgrounds and with a range of needs, should occur throughout the full development cycle, from design to implementation and evaluation. By positioning young people as active partners in decision-making, who often have a deeper understanding of digital environments than the adults shaping research, practice, and policies, outcomes can better reflect their needs and promote responsible and informed use of digital technologies.

Digital mental health tools that fail to reflect how young people actually use technology—or how they interpret and adopt these tools—risk being harmful, irrelevant, or ineffective [17]. In a context where young people are already turning to digital and AI-based tools for mental health support, the absence of young people’s voices in research and product design represents a critical blind spot. The participation gap is both a practical and rights-based concern, as excluding young people from digital mental health research risks undermining innovation quality and the United Nations (UN)–recognized rights of children in digital contexts [18]. As such, efforts to develop and improve standards and practices for digital mental health are urgently needed.

As noted, the rapid emergence of GenAI further intensifies the risks associated with youth exclusion from digital mental health research. These systems are also highly scalable and increasingly embedded in everyday platforms, meaning that design decisions made without youth input can quickly affect large populations. In this context, the exclusion of young people is not only a matter of representation but also a potential source of harm.

**Figure 1:**
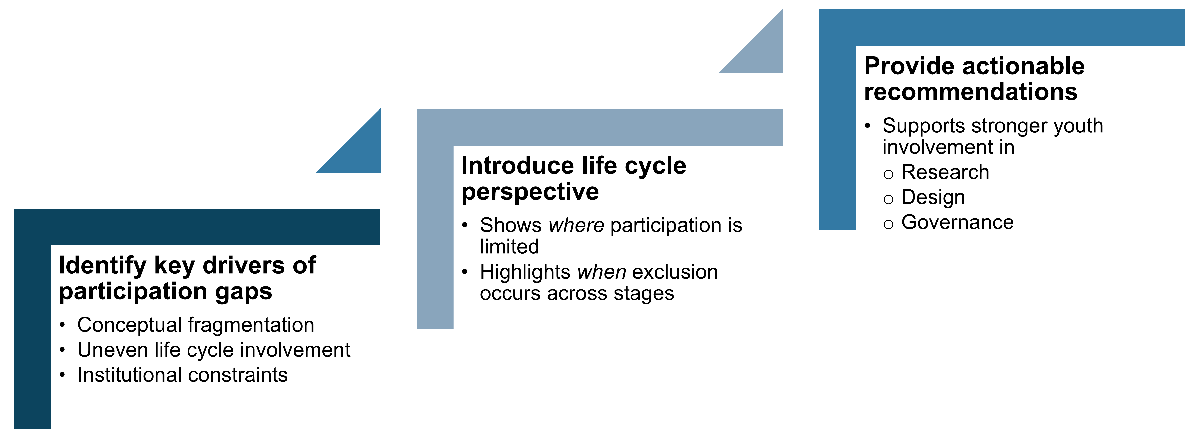
Summary of Viewpoint aims.

This Viewpoint examines the persistent gap between the widespread endorsement of youth participation in digital mental health research and its inconsistent implementation in practice. It advances 3 contributions (Figure 1). First, it surveys the key drivers of this participation gap, including conceptual fragmentation, uneven involvement across the research life cycle, and institutional constraints. Second, it introduces a life cycle–oriented perspective to highlight where and how youth participation is most often limited or excluded. Third, it sets out a series of concrete, actionable recommendations to support more consistent and meaningful youth involvement across research, design, and governance.

The intended audience for this Viewpoint includes mental health researchers, human-computer interaction (HCI) and design researchers, ethics committees, funders, journal editors, and developers of digital and GenAI-enabled mental health tools. By clarifying both the nature of the problem and practical steps for addressing it, this paper aims to contribute to a more coherent and accountable approach to youth participation in digital mental health.

## Conceptual Fragmentation in Youth Participation

A recurring challenge in DYMH research is the lack of conceptual coherence in how participatory approaches are described and operationalized. Reviews of youth participation highlight substantial variability in terminology, with concepts such as co-design, coproduction, PPI, participatory research, and user-centered design frequently used interchangeably despite reflecting different assumptions about influence, power sharing, and decision-making authority [5].

In this Viewpoint, we use *youth participation* as an umbrella term to describe approaches that involve young people in shaping research and intervention development. Within this, we distinguish between several commonly used but conceptually distinct approaches. *User-centered design* typically positions young people as informants whose needs and behaviors are elicited and interpreted by researchers to improve usability and fit. *Participatory design* and related HCI approaches emphasize more active involvement, with users contributing to design processes and decision-making. *Co-design* is used here to denote more sustained and iterative collaboration, in which young people contribute to shaping research priorities, design decisions, and evaluation. *PPI*, rooted in health research, focuses on incorporating lived experience into research processes, often with an emphasis on accountability and relevance.

While these approaches overlap in practice, they differ in their assumptions about influence, power sharing, and the role of young people. Inconsistencies in how these terms are used and reported can obscure the extent and nature of youth involvement, making it difficult to assess what participation entails in practice. We recognize that definitions and applications of co-design vary widely across studies, as documented in reviews of DYMH research [4]. In DYMH, meaningful co-design can strengthen intervention relevance, acceptability, and engagement, while also supporting young people’s empowerment and skills through more egalitarian youth-adult partnerships and shared decision-making [19-23].

Originating in early work on “software psychology” [24], HCI foregrounded the importance of human behavior in system design, largely to improve usability and user experience. Work within HCI and child-computer interaction has now developed more granular taxonomies to distinguish between participatory approaches. These include distinctions between user-centered design, participatory design, and co-design, as well as traditions such as cooperative inquiry, which explicitly position children and young people as active design partners rather than passive informants.

Such frameworks differentiate approaches based on the degree of user influence, stages of involvement, and underlying assumptions about power sharing. Despite this, these distinctions are not consistently applied or reported in digital mental health research, contributing to ongoing conceptual ambiguity. In particular, the cooperative inquiry tradition within child-computer interaction [25,26] has developed well-established methods for involving children and young people as active design partners rather than passive informants. These approaches emphasize sustained engagement, iterative cocreation, and shared decision-making, directly addressing challenges related to power imbalances and tokenistic participation. Such methods demonstrate that meaningful youth involvement is both feasible and methodologically robust. However, these approaches remain underused and inconsistently translated into digital mental health research, where participation is more often limited in scope or duration.

By contrast, psychological intervention development has typically prioritized youth involvement to strengthen the acceptability and relevance of therapeutic content and its capacity to change cognition and behavior. As digital technologies increasingly integrate technical and therapeutic elements, these traditions have converged, but overlapping terms (eg, *co-design*, *user-centered design*, *user experience*, and *PPI*) continue to carry discipline-specific meanings and functions. As a result, the same participatory activity may be framed as optimizing technical usability, enhancing therapeutic relevance, or both, limiting clarity about what participation is intended to achieve and how young people’s influence is enacted in practice.

We suggest, at the outset, that without clearer conceptual alignment and explicit reporting of participatory models, language around participation risks functioning as a proxy for good practice rather than as evidence of meaningful youth partnership.

## Different Models of Participation and the Problem of Life Cycle Gaps

A further and related challenge in DYMH research is the coexistence of multiple models of youth participation, often applied inconsistently and without explicit justification. This problem may be compounded by disciplinary research divides and research grant funding constraints [27]. Frameworks such as Hart’s ladder of participation [28], Lundy’s rights-based model [29], and, specific to mental health, the 4-L framework, which recognizes the value of lived, loved, labored, and learned experience in research [30], offer distinct ways of conceptualizing participation, ranging from consultation and lived experience to shared decision-making and youth-led action [31,32]. In HCI, co-design and participatory methods often treat participation as a continuum, yet studies frequently fail to justify their chosen approach or reference underlying frameworks [31]. However, DYMH studies also rarely specify which model they are drawing on or how participation is expected to function in practice.

Lundy’s model, grounded in Article 12 of the UN Convention on the Rights of the Child, is particularly influential in framing participation as a rights-based process rather than a methodological choice [29]. It emphasizes 4 core components—*space, voice, audience,* and *influence*—highlighting that simply giving young people an opportunity to speak is insufficient if their views are not heard or acted upon [29]. This distinction is critical in digital mental health research, where youth “voice” is often solicited through surveys or workshops [33], but decision-making authority remains firmly with adult researchers, clinicians, or developers.

A related issue concerns how the “success” or quality of participation is defined and evaluated. While frameworks such as GRIPP2 (Guidance for Reporting Involvement of Patients and the Public) [34] provide structured approaches for reporting PPI in health research, their application in DYMH remains inconsistent. In particular, existing reporting standards often focus on documenting activities rather than assessing the extent of influence, power sharing, or impact on research outcomes. As a result, there is limited consensus on how to distinguish between tokenistic and meaningful participation or how to compare participatory practices across studies.

While the Lundy model of participation [35] and associated checklists [36] offer more explicit criteria for evaluating the quality of participation, including considerations of voice, influence, and feedback, these tools are not routinely embedded within DYMH research practice. In parallel, practical resources such as peer researcher training programs could offer guidance on meaningful youth involvement across the research life cycle, particularly during implementation and evaluation phases.

More generally, there remains limited consensus on what constitutes high-quality, youth-centered participation in digital mental health research or how it should be embedded consistently across the research life cycle [5,37]. In mental health research, there have been checklists developed to support best practice guidance and reporting on PPI [38], which can inform PPI practices in DYMH, and there is growing interest in this topic in DYMH [5]. These differences also reflect distinct disciplinary origins. PPI is largely rooted in the United Kingdom and European health research policy, emphasizing accountability and lived experience.

Other concerns relate to the synthesis of the evidence base used to identify best practices, which, as previously noted, depends on the models of youth participation that are invoked. For example, human-centered design and user-centered design typically position young people as informants whose needs and behaviors are elicited and interpreted by researchers, while participatory design emphasizes empowering users to influence decision-making [31,39]. Co-design goes further by positioning them as active partners in generating ideas, prototyping solutions, and shaping outcomes [32]. PPI involves a collaboration between researchers and young people, in which lived experience informs decisions throughout the research and intervention process [40]. This approach promotes shared decision-making and integrates diverse perspectives, ensuring digital tools and mental health programs are relevant, credible, and responsive to the needs of young people [40].

Relatedly, participatory work on youth empowerment stresses that meaningful involvement requires shifts in power, culture, and institutional norms, not merely the addition of participatory activities [19,23]. Adult-centric research cultures—characterized by paternalism, risk aversion, and assumptions about young people’s capacity—continue to shape how participation is designed and constrained. These norms risk relegating young people to episodic or symbolic involvement rather than positioning them as sustained partners across the research life cycle.

Life cycle gaps are particularly evident in DYMH [5]. Young people may be involved at early ideation stages or during usability testing, yet excluded from problem formulation, theory selection, implementation decisions, or evaluation of impact [20]. This problem is even more prominent among marginalized and disadvantaged groups [41]. This selective involvement undermines claims of co-design and limits the potential of participation to shape outcomes meaningfully. As a result, there is a risk that DYMH interventions co-designed with youth revert to organizationally driven design processes that lack meaningful youth involvement at a later stage. Without explicit attention to *where* and *how* participation occurs across the life cycle and without consistent reporting across studies, youth involvement risks becoming overlooked, superficial, or disjointed.

Addressing this limitation requires approaches that move beyond late-stage usability testing, which often positions young people as “test subjects” of already prototyped or predefined interventions. Instead, co-design should incorporate creative, youth-driven methods that support earlier and more meaningful engagement with ideas [42,43]. These include approaches such as illustration, digital storytelling, play-based activities, and the use of simple analogies to represent system functions [44]. Such methods can help translate abstract or technical concepts into developmentally appropriate forms, enabling young people to engage more critically with the design of digital tools.

Taken together, these limitations point to the need for an integrated participatory framework—one that combines rights-based models of youth participation with design-oriented methodologies and clinical insight. At a minimum, researchers should clearly state which participation model they are adopting, how it informs youth involvement across the research life cycle, and how young people’s influence is enacted in practice. Without such clarity, claims of co-design risk reinforcing adult-centric norms within research practices rather than challenging them.

## Institutional Barriers That Keep Youth at the Margins

Even when there is a commitment to youth involvement, institutional and governance barriers limit meaningful participation in digital mental health research. These constraints operate upstream of study design and frequently determine whether youth coproduction is feasible in practice. The practical barriers described in this section—including fragmented reimbursement processes, sectoral misalignment with youth organizations, and variable researcher confidence in participatory methods—reflect recurring themes raised in preliminary stakeholder consultations conducted as part of the YouthDMH Delphi (CA23153) COST Action [45]. These are presented here as propositions for the field rather than as established findings and are being formally examined in that ongoing process.

Ethical review processes may pose a major obstacle [46,47]. As identified in our own findings, research ethics committees may express uncertainty when evaluating participatory research and PPI with children and adolescents, particularly in digital and mental health contexts. Heightened concerns about safeguarding, data protection, and psychological risk could lead to sampling that limits the inclusion of the very populations the technology is designed for, constraining the generalizability of findings. In response, participatory elements may be deliberately scaled back or excluded—not because they are unethical but because they are perceived as challenging to govern, creating additional burdens for health researchers. Development of ethical guidelines for the participation of young people in DYMH research is needed, aligning with existing frameworks such as Orygen’s Youth Partnerships in Research Toolkit [48]. There is also a need to embed youth participation as a component of research within organizations so that youth co-design is not merely a one-time event but is continuously integrated into projects. This involves targeted training for clinicians, youth workers, and other professionals working in clinical or youth organizations to build confidence in participatory approaches and to better articulate the value of youth involvement in improving research relevance and impact on services and policy.

Consent requirements introduce further friction [46]. Variability in age-of-consent thresholds, combined with requirements for parental or guardian permission alongside youth assent, creates perceptions and realities of administrative and ethical complexity [49]. Flexible, tiered models of consent—where lower-risk activities rely primarily on youth assent and higher-risk engagements require additional parental involvement—may help to reduce barriers while maintaining safeguarding standards [46]. Without such adaptations, current consent practices risk reinforcing existing inequalities in access and undermining the inclusivity of youth engagement. Current arrangements may risk unintentionally excluding young people with limited parental support or those seeking confidential mental health help, while also discouraging sustained engagement beyond one-off, tokenistic consultations.

Financial and administrative uncertainty compounds these challenges. Many researchers in our YouthDMH Delphi poll (CA23153) identified a lack of clear guidance on reimbursing young people appropriately, generating anxiety about coercion and risk [45]. One concern is that reimbursement processes are frequently fragmented across institutions, creating practical barriers to consistent and meaningful youth involvement. Youth participation is rarely adequately costed in research grants and can also be treated as optional rather than essential, despite young people’s recommendations [50]. Hyperspecialization and grant culture have been identified as major influences on research outcomes [27], and participants in our Delphi research identified short funding cycles and pressure for rapid outputs as further undermining slower, steadier relational work required for meaningful engagement with younger people. Sectoral misalignment may also play a role. Youth charities and nongovernmental organizations are central to support and advocacy, but our survey participants suggested that they are not always structured or resourced to engage in timely research partnerships. Where collaboration exists, participants worried that service delivery priorities may take precedence over research participation.

Another barrier may arise from researchers’ limited understanding of the value of including youth in co-design throughout the research process [21,37]. Challenging the perception that expertise exists only in academic or professional forms is essential. Relatedly, as our Delphi participants noted, meaningful youth participation requires additional time, and flexibility must be embedded within research timelines to support sustained engagement.

It should also be emphasized that these barriers may not be uniform across contexts. In lower-resource and Global South settings, additional constraints such as digital exclusion, limited infrastructure, and differing regulatory or cultural norms may further shape opportunities for youth participation [51,52]. Intersectional factors—including socioeconomic disadvantage, marginalization, and mistrust of institutions—may also limit whose voices are included, raising the risk that participatory approaches reproduce existing inequities if not carefully adapted [41,51,52]. Together, these institutional barriers may risk reinforcing entrenched norms that prioritize risk management over youth agency. Without addressing them directly, calls for greater youth involvement in digital mental health research will remain largely aspirational.

A final and urgent challenge is that most GenAI tools used by young people are developed and deployed by large, proprietary technology companies, whose design processes are not subject to the same participatory expectations as academic or publicly funded health research [53]. Companies such as OpenAI and Google operate at a scale and speed, and their commercial incentives—often centered on engagement, retention, and market expansion—may collide with youth well-being or safeguarding priorities [52,54].

This creates a structural asymmetry: young people are among the most intensive users of GenAI-enabled systems, yet they have little to no influence over how these systems are designed, governed, or iteratively updated [54,55]. In contrast to traditional digital mental health interventions, which are often developed within research or clinical contexts where participatory approaches can be embedded, GenAI tools are frequently introduced into everyday environments before participatory safeguards or evidence-based frameworks are established [56,57]. Addressing this gap requires expanding the concept of participation beyond individual studies to include system-level accountability. This may include legislative and regulatory expectations for youth-informed safety standards, independent oversight mechanisms, and the integration of youth advisory structures into industry-academic partnerships. Without such co-design approaches, we suggest that calls for co-design risk remaining confined to small-scale research settings, while the most widely used systems continue to evolve without meaningful youth input.

## Recommendations

**Figure 2:**
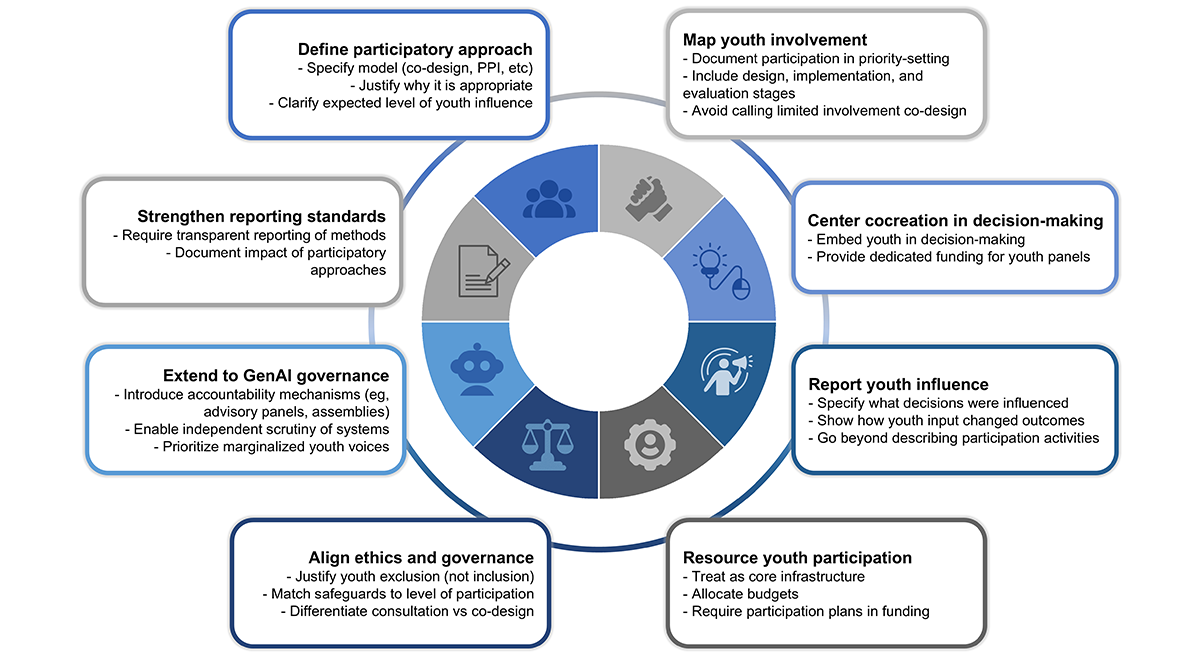
Recommendations. GenAI: generative artificial intelligence; PPI: patient and public involvement.

Closing this gap requires moving beyond general commitments to inclusion toward concrete, implementable practices. We propose a set of minimum, normative expectations to support meaningful youth participation across research, design, and implementation (these are summarized in Figure 2).

First, we recommend that participatory approaches be explicitly specified and justified [34]. Researchers should clearly state which model (eg, co-design, PPI, and participatory design) is being used, why it is appropriate, and what level of influence young people are expected to have [34].

Second, youth involvement should be mapped across the research life cycle [5,28]. Studies should document where and how young people are involved, including priority setting, intervention design, implementation, and evaluation. Involvement limited to early ideation or usability testing should not be presented as co-design.

Third, cocreation should be central—panels involving youth should receive dedicated funding and be embedded into decision-making processes on youth and digitalization, ensuring that young people’s perspectives are systematically heard and acted on [16,29,35]. Studies should report youth influence, not just participation. Beyond describing participatory activities, researchers should specify what decisions young people influenced and how their input changed the research or intervention.

Fourth, youth participation must be resourced as core research infrastructure [30]. Funders and institutions should require dedicated and justified budget lines for participatory work, for example, through minimum allocation thresholds or explicit justification where such costs are absent. We urge that participation plans, including timelines and roles for young people across the research life cycle, be assessed as part of funding decisions.

Fifth, governance and ethical oversight should be aligned with youth agency. Ethics committees should adopt proportionality-based approaches that require researchers to justify the exclusion of young people rather than their inclusion [47,48]. Review processes should distinguish between different forms of participation (eg, consultation vs co-design) and assess whether safeguarding measures are proportionate to the level of involvement. Where participation is limited or absent, researchers should be required to provide a clear rationale.

Sixth, participation must be extended to the governance of GenAI systems [14]. As noted, many tools used by young people are developed by proprietary technology companies such as OpenAI and Google. This creates a fundamental limitation: the systems most widely shaping young people’s mental health experiences are not designed within participatory research frameworks.

Rather than assuming that traditional models of co-design can be directly applied, we recommend that this gap be addressed through mechanisms of accountability. This includes independent scrutiny of system behavior, clearer standards for youth safety, and structured ways of incorporating young people’s perspectives into evaluation and governance processes. Such approaches may include youth advisory panels, deliberative forums, or youth citizen assemblies that can inform regulatory, ethical, and design standards at a system level, even where direct participation in development is not possible [58]. Here, we should ensure the active involvement of people from marginalized communities and young people with lived experiences in policy development and regulation to reduce bias and discrimination associated with these tools [52]. Without such measures, young people risk being positioned as passive recipients of systems that influence how they interpret distress, seek support, and engage with mental health content. Finally, we recommend that journals and funders strengthen reporting expectations. Requiring transparent reporting of participatory methods and their impact would improve consistency, comparability, and accountability across the field. In the context of rapidly evolving digital and GenAI technologies, these steps are essential to ensure that innovation is not only scalable but also safe, credible, and responsive to the needs of young people.

We emphasize that the previously stated recommendations are not intended as definitive but as a foundation for further empirical refinement. As part of the COST Action YouthDMH (CA23153), the authors are members of a dedicated working group focused on advancing youth participation in digital mental health [45]. This work is currently being extended through a multistakeholder Delphi process involving young people, researchers, clinicians, parents, and policymakers, with the aim of developing consensus-based guidance for meaningful youth involvement across digital and GenAI-enabled mental health research. A structured reporting checklist and life cycle mapping template are currently being developed as companion outputs of an ongoing multistakeholder Delphi process and will be published separately.

## Conclusions

This Viewpoint aims to bring updated and renewed attention to a persistent disconnect in DYMH research: young people are among the most intensive users of digital and GenAI-enabled tools, yet they remain marginal to the research and design processes that shape these technologies. The participation gap is sustained by conceptual ambiguity, uneven involvement across the research life cycle, and institutional barriers that collectively render youth participation difficult, episodic, or symbolic rather than routine and influential. In line with Mental Health Europe [15], we advocate for a cocreation approach and a human rights framework to serve as the compass for any developments in digital mental health. Only with this collaborative approach—in every step of the process, from design to evaluation—can digital technologies align with real needs and work toward realizing a vision of a society where everybody can fully enjoy their human rights and thrive.

## Abbreviations

AI: artificial intelligence
DYMH: digital youth mental health
GenAI: generative artificial intelligence
GRIPP2: Guidance for Reporting Involvement of Patients and the Public
HCI: human-computer interaction
PPI: patient and public involvement
UN: United Nations
